# Reply to: Investing in equitable healthy aging: Why Africa must reform social pension schemes to improve Alzheimer's disease and dementia outcomes

**DOI:** 10.1002/alz.70298

**Published:** 2025-05-19

**Authors:** Josephine E. Prynn, Sebastian Walsh

**Affiliations:** ^1^ King's College London London UK; ^2^ Cambridge Public Health University of Cambridge Cambridge UK

1

Dear Editor,

The paper from Mostert et al.^1^ makes a strong and important case for the potential of universal pensions to enhance equitable and healthy aging in all global contexts. We agree with many of the arguments presented, including the overarching desire for social justice and evidence‐based public policy to improve health in old age. However, we noted four key issues/omissions that we consider will be helpful to readers as they consider the implications of this thought‐provoking perspective piece; these are described in detail below.

First, one of the central arguments of Mostert et al.^1^ is based upon a comparison between the age of eligibility for pensions and life expectancy at birth across multiple African settings. But life expectancy at birth is highly influenced by mortality in early years, which unfortunately remains unacceptably high in many settings. A more informative impression of the number of people reaching older age and their likely age at death is life expectancy at age 45–that is, the years a person is expected to live once they have survived childhood and early adulthood.

Life expectancy in Africa at birth is 63.8 years, but life expectancy at age 25 is 70.7 years, and at age 45 is 73.8 years.^2^ Therefore, the assertion that “most African men are dead” at the time of first pension payouts at age 63 is perhaps disingenuous; if they have survived childhood and early adulthood, most people will still be alive at the age of 63, with an average of 10.8 years left of life.

Further, the paper asserts that “The pension systems in Botswana, Lesotho, Mozambique, Namibia, Eswatini, Nigeria, Kenya, Tanzania, and Uganda are structured such that men may only benefit from the pension pay‐outs for a minuscule period (less than a year).” However, considering life expectancy at 45, in all but Lesotho, most people would be expected to be eligible for a pension in the last 4 to 14 years of their lives, as shown in Table 1.

**TABLE 1:** Pension age, life expectancy at birth, and life expectancy at age 45 for a selection of African countries.

Country	Pension age	Life expectancy at birth (2023)	Life expectancy at age 45 (2023)
Botswana	W 65 M 65	69.2	75.1
Lesotho	W 70 M 70	57.4	69.2
Mozambique	W 55 M 60	63.6	72.4
Namibia	W 60 M 60	67.4	74.1
Eswatini	W 60 M 60	64.1	72.6
Nigeria	W 65 M 65	54.5	70.9
Kenya	W 65 M 65	63.6	71.9
Tanzania	W 70 M 70	67.0	73.9
Uganda	W 65 M 65	68.3	75.0

*Source*: Our world in data^2^

Second, there is perhaps a slightly rose‐tinted presentation of pension policies in European countries. In the UK, for example, the pension age is 67. But only the most affluent 20% of men and 30% of women have a healthy life expectancy that exceeds this.^3^Though, as the authors note, Africa is many decades away from the demographic distribution experienced in Europe, systems should be designed for long‐term sustainability and resilience to both demographic change and unexpected crises.

Third, while there is some discussion about the affordability of expanding social pension schemes in Africa, there is no consideration of opportunity cost. All countries face unlimited wants with limited resources. By increasing public spending on option A, benefits that would have been enjoyed by spending those resources on option B are foregone. An example might be dementia care, the cost of which will increase exponentially as Africa and the global population age. Though the authors describe some compelling benefits seen in the health of older people since the 2008 pension reforms in South Africa, much of the evidence linking pensions to health benefits is based on imperfect and complex data from which extracting specific and robust causal estimates is difficult. Therefore, policy makers need to weigh possible health gains from expenditures on pension expansion against, for example, increasing resources available to provide de‐stigmatized, holistic care for people living with dementia and other diseases of older age.

Finally, some consideration of the factors behind the figures presented would be welcome. Why is life expectancy more than a decade longer in Botswana than in Lesotho? Why do women have longer healthy life expectancy than men, and why is this difference more profound in some countries than others? The answers point to upstream, social determinants that affect health cumulatively across the life course. These are also opportunities for public policy improvements that can ultimately help to achieve healthy aging and reduce dementia morbidity—noting, again, that such investments would be potential opportunity costs of pension expansion.

None of this is to say that enhancing access to pensions in older age in African settings is not important or urgent—it is both. But, particularly in settings in which resources are scarce and there is far from universal coverage from existing pension schemes, planning needs to be based on a robust understanding of the data to ensure that those with the most critical health and social needs are prioritized, to promote equitable and healthy aging.

## CONFLICT OF INTEREST STATEMENT

The authors declare no conflicts of interest. Author disclosures are available in the supporting information.

## Supporting information



Supporting Information
